# Enhanced Biological Activity of a Novel Preparation of *Lavandula angustifolia* Essential Oil

**DOI:** 10.3390/molecules26092458

**Published:** 2021-04-23

**Authors:** Małgorzata Miastkowska, Tomasz Kantyka, Ewa Bielecka, Urszula Kałucka, Marta Kamińska, Małgorzata Kucharska, Anna Kilanowicz, Dariusz Cudzik, Krzysztof Cudzik

**Affiliations:** 1 Department of Chemical Engineering and Technology, Cracow University of Technology, Warszawska 24, 31-155 Cracow, Poland; 2 Malopolska Centre of Biotechnology, Jagiellonian University, 30-387 Kraków, Poland; tomasz.kantyka@uj.edu.pl (T.K.); ewa.bielecka@gmail.com (E.B.); urszula.kalucka@student.uj.edu.pl (U.K.); marta_kaminska@outlook.com (M.K.); 3 Department of Toxicology, Faculty of Pharmacy, Medical University of Lodz, Muszynskiego 1, 90-151 Lodz, Poland; malgorzata.kucharska@umed.lodz.pl (M.K.); anna.kilanowicz@umed.lodz.pl (A.K.); 4 Ostrowieckie Centrum Medyczne Civil Partnership Anna Olech-Cudzik, Krzysztof Cudzik Iłżecka 31A, 27-400 Ostrowiec Świętokrzyski, Poland; dariuszcudzik@cudmed.pl (D.C.); krzysztofcudzik@cudmed.pl (K.C.)

**Keywords:** *Lavandula angustifolia* essential oil, GC/MS analysis, wound healing, proinflammatory cytokines, proregenerative growth factors, HaCaT, VEGF

## Abstract

*Lavandula angustifolia*, one of the most popular medicinal plants, is the source of a bioactive essential oil characterized by a wide spectrum of biological activity, e.g., antiseptic, analgesic, and anticancer effects. In dermatology, the oil helps to relieve skin inflammation and exhibit wound healing potential. However, the mechanism of action of the lavender oil depends on its composition, which in turn is dependent on the origin and growing conditions. Our study aimed to compare the composition and proregenerative properties of the commercially-available narrow-leaved lavender oil produced in Provence, France, with the oil obtained from the narrow-leaved lavender cultivated locally in Poland. GC/MS analysis showed that self-manufactured essential oil had lower linalool content than commercial oil (23.2 vs. 40.2%), comparable linalyl acetate content (40.6 vs. 44%), while the proportion of lavandulyl acetate was significantly higher (23.2 vs. 5.5%). To determine the influence of lavender oil on the production of proinflammatory cytokines and proregenerative growth factors, gene expression of the selected signaling molecules by HaCaT cells was investigated using real-time PCR. Results showed a concentration-dependent effect of lavender oils on the production of IL-6, IL-8, and VEGF by the keratinocyte cell line. Finally, the potential of the lavender oil to increase the production of VEGF, the most important angiogenic factor, with the in-house preparation performing significantly better in the in vitro cell models was identified.

## 1. Introduction

Lavender is one of the most popular plants in the world and originates from the mountain areas of the Mediterranean. The Lavandula genus belongs to the *Lamiaceae* family and includes over 30 species, out of which the most commonly cultivated are: *L. angustifolia Mill*. (narrow-leaved lavender), *L. stoechas* (French lavender) oraz *L. latifolia* (broad-leaved lavender). Its generic name comes from the verb lavare, which means “to wash” in Latin [1]. Out of all Lavandula genera, the most valuable genus in terms of unique biological activity is narrow-leaved lavender (*Lavandula angustifolia*, formerly *L. officinalis Chaix* or *L. vera*), also known as garden lavender [2,3].

Lavender oil is distilled from the flowers and the top growth of narrow-leaved lavender [4]. The medicinal raw material comes mainly from the flowers, with the essential oil obtained ca. 3%. The essential oils from the Lavandula genus contain similar components but in different proportions. The quality and composition of the essential oil depend on the origin, growing conditions, variety, and other factors. Lavender oil can contain more than 100 different components. Mainly, these are terpenoid compounds (monoterpenes, triterpenes, sesquiterpenes) as well as phenolic compounds (flavonoids, phenolic acids, coumarin, tannins). The main components of the essential oil should be linalool (20–45%) and its acetate (25–46%). The percentage of other ingredients falls usually within the following ranges: limonene (<1.0%), eucalyptol (<2.5%), camphor (<1.2%), terpinen-4-ol (0.1–6.0%), lavandulol (>0.1%), lavandulyl acetate (>0.2%), and α-terpineol (<2.0) [2,5,6].

Lavender oil is characterized by a variety of pharmacological activities ranging from anti-inflammatory to antioxidant, antibacterial, antifungal, antiseptic, antiviral, antidepressive, sedative, immune-stimulating, and even anticancer effects [1,7]. It has a strong bactericidal effect on many strains of bacteria (e.g., *Staphylococcus aureus, Enterococcus faecalis*), accompanied by a weak fungicidal activity (e.g., *Botrytis cinereal*, *Aspergillus fumigatus*). Formulations containing lavender oil are used to treat inflammation in the mouth and throat and upper respiratory tract infections. In dermatology, the essential oil preparations help to relieve skin inflammation, psoriasis, or eczema [1]. Moreover, the oil is a component of ointments used to treat wounds and burns. According to the literature, pure lavender oil was used to treat wounds after burns during World War I [1]. Due to its antimicrobial properties, it causes the purulent states of the skin to subside and it improves the healing rate of the wounds. It should be noted, however, that the direct contact of essential oils with the skin can lead to irritation and allergic reactions [8]. In aromatherapy, lavender oil acts as a remedy with a potential sedative, sleeping, anxiolytic, and mood-enhancing effect [4]. In the cosmetic industry, it is used to produce bath salts, shampoos, skin tonic, cosmetic face masks, and for aromatizing naturally manufactured creams, balsam, or toilet waters [9]. The essential oil may also be used to preserve cosmetic products as an effective alternative for synthetic preservatives such as DMDM hydantoin, which acts as formaldehyde releaser [10].

Lavandula genus is currently the subject of genetic engineering, as a result of which new variants of garden lavender are being created. The plant contains various biologically active substances which have therapeutic potential, but there is a lack of relevant information on the dosage of lavender preparations. Currently, further research is underway on the mechanisms of the effects of lavender and its metabolites and the safety of their use. The majority of research is focused on studying and comparing bacteriostatic and antioxidant properties of different lavender varieties [3,11,12,13].

The purpose of this study was to assess the effect of self-manufactured and commercially available lavender oil on the proinflammatory and proregenerative effect of human keratinocytes in the HaCaT model. In addition, the qualitative and quantitative composition of the two oils was compared.

## 2. Results

### 2.1. Identification of Lavender Oils

#### 2.1.1. Qualitative Analysis of Essential Oils

**Figure 1:**
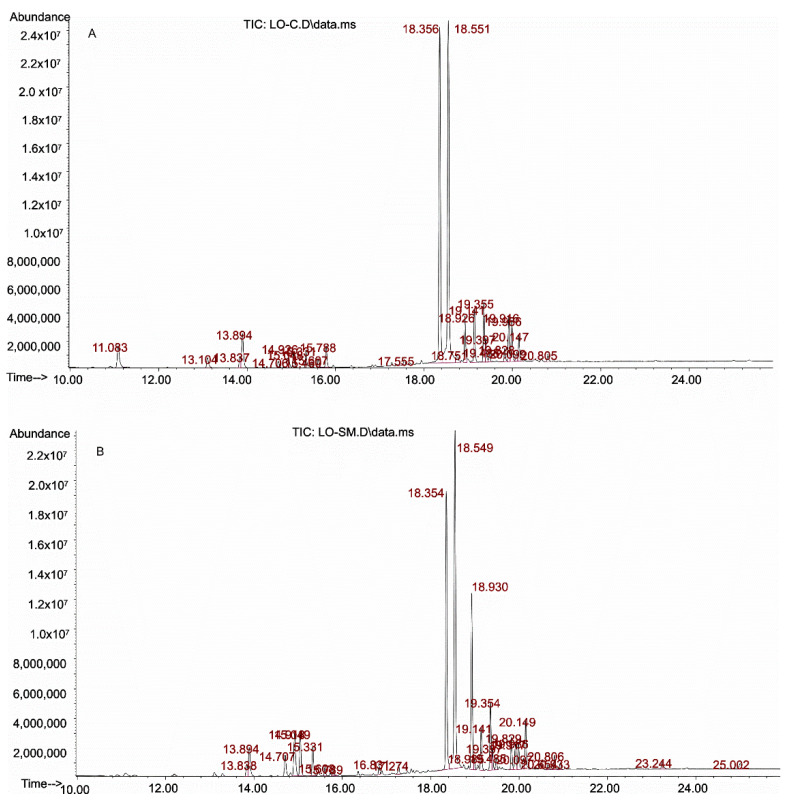
Chromatograms of the tested samples of lavender oils: **A**—commercial sample (LO-C), **B**—self-manufactured sample (LO-SM).

The chromatogram of the commercial lavender oil sample (LO-C) allowed for the identification of 35 chromatographic peaks (Figure 1A), of which 17 were peaks with a significant proportion (over 1% of the total peak area). The main identified peaks were respectively: linalool, representing 28.1% of the share in the chromatogram; and linalyl acetate (bergamol), with a share of 30.1%. Other substances identified in this sample were: caryophyllene—4.2% share, β-myrcene—3.9%, terpinen-4-ol—3.7%, lavandulyl acetate—3.4%, borneol 3.0%, neryl acetate—2.8%, and α-pinene—2.5%. Among others, constituting 1–2% of the sample, the following substances should be mentioned: β-pinene, eucalyptol (1,8-cineole), *cis*- and *trans*-ocymene, *o*-cymene, camphor, β-fernesene, and geraniol acetate.

In the tested self-manufactured lavender oil—LO-SM (Figure 1B), chromatographic analysis allowed for the identification of 49 peaks, 14 more than in the commercial preparation. Fifteen of them were peaks with a significant proportion (>1%). Similar to the commercial preparation of the lavender oil, linalool and linalyl acetate were the dominant peaks, although their share was smaller than in the previous sample—18.5 and 26.0%, respectively. Lavandulyl acetate was present in a significant amount—11.0%, when compared to the commercial sample (3.4%). Other identified substances with a >2.0% share in the chromatogram peak area were: caryophyllene—4.0%, geraniol acetate—3.5%, eucalyptol 3.4%, *trans*-ocimene—2.8%, terpinen-4-ol—2.7%, β-mircen—2.3%, borneol—2.0%, and α-terpinol—2.0%. The remaining substances with a share of 1–2% in the chromatogram were D-limonene, *cis*-ocymene, β-fernesene, and neryl acetate,

On the basis of the identification analysis, the substances with the highest share in the analyzed samples were selected for quantitative research and included: linalool, linalyl acetate and lavandulyl acetate, as well as substances characteristic of lavender growing in Poland, i.e., caryophylene, bornyl acetate, and geraniol [6].

#### 2.1.2. Quantitative Analysis

Standard solutions of test substances were prepared by dissolving the appropriate amounts of compounds in dichloromethane and subsequently served to prepare the calibration curves. The range of the determined concentrations was about 500 ÷ 4000 µg/mL for linalool and linalyl acetate, for lavandulyl acetate—100 ÷ 1000 µg/mL, and for other substances—50–500 µg/mL. The validation parameters for individual substances are presented in Table 1. Determined concentrations were then converted to the percentage (*w*/*w*) content and results are presented in Table 2.

As the presented quantitative analyzes show, the tested oil (LO-SM) differed from commercial oil. LO-SM oil had lower linalool content than LO-C oil (23.2 vs. 40.2%) and comparable linalyl acetate content (40.6 vs. 44.0%), while for lavandulyl acetate, this content was significantly higher (23.2 vs. 5.5%). In the case of substances present in trace amounts, the differences were smaller, although the share of (−)-*trans*-caryophyllene and (−)-geraniol was greater in the analyzed oil (LO-SM) than in commercial oil. No bornyl acetate was found in both oils, while isobornyl acetate was identified in trace amount.

### 2.2. Lavender Oil Cytotoxicity

**Figure 2:**
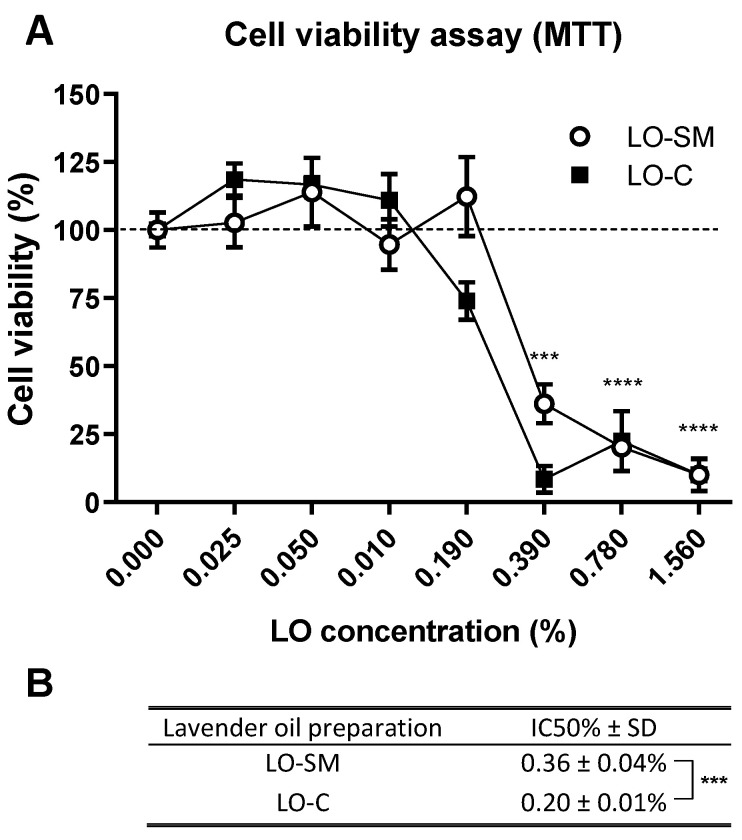
Cytotoxicity of tested lavender oils. (**A**) Cell viability assay (MTT) was performed after 24 h incubation of HaCaT cells with designated concentrations (*v*/*v*) of commercial or in-house preparation of lavender oil. Values are mean ± SD. Groups were compared to the control, and statistical analysis was performed using GraphPad Prism and build-in ANOVA test. *p*-values ≤ 0.05 were considered significant. ***—*p* ≤ 0.001; ****—*p* ≤ 0.0001. (**B**) IC50% values were interpolated as lavender oil concentration required for the 50% reduction in cell viability. The data represent mean ± SD between four replicates. Statistical significance was evaluated using a *t*-test. ***—*p* ≤ 0.001.

The maximum concentration of lavender oil was optimized in the preliminary experiments, where up to 25% concentration was investigated. For the present study on full lavender oil, we observed complete cytotoxicity at 1.5% concentration, and these conditions were chosen for further evaluation and IC50% determination, as shown in Figure 2. Serial dilutions were prepared in the cell culture medium from 25% of each preparation to 0.025% and were incubated with HaCaT cells for 24 h. The cell viability was then estimated by formazan crystal deposition and absorbance measurement at 570 nm. The results of the control cells incubated in the culture medium alone were used for the comparison and represented 100% cell viability. The general cytotoxicity of the oils was high, and already 0.4% LO-C caused a reduction of cell viability to 10% of the control sample, compared to the moderate reduction (36% of the control cell viability) for the same concentration of commercial preparation LO-SM (Figure 2). The 0.1% concentration was identified as safe for all tested preparations and was employed as the high concentration limit in all subsequent cell-based experiments.

### 2.3. Effect of Lavender Oil Preparations on the Cytokine Cellular Response

**Figure 3:**
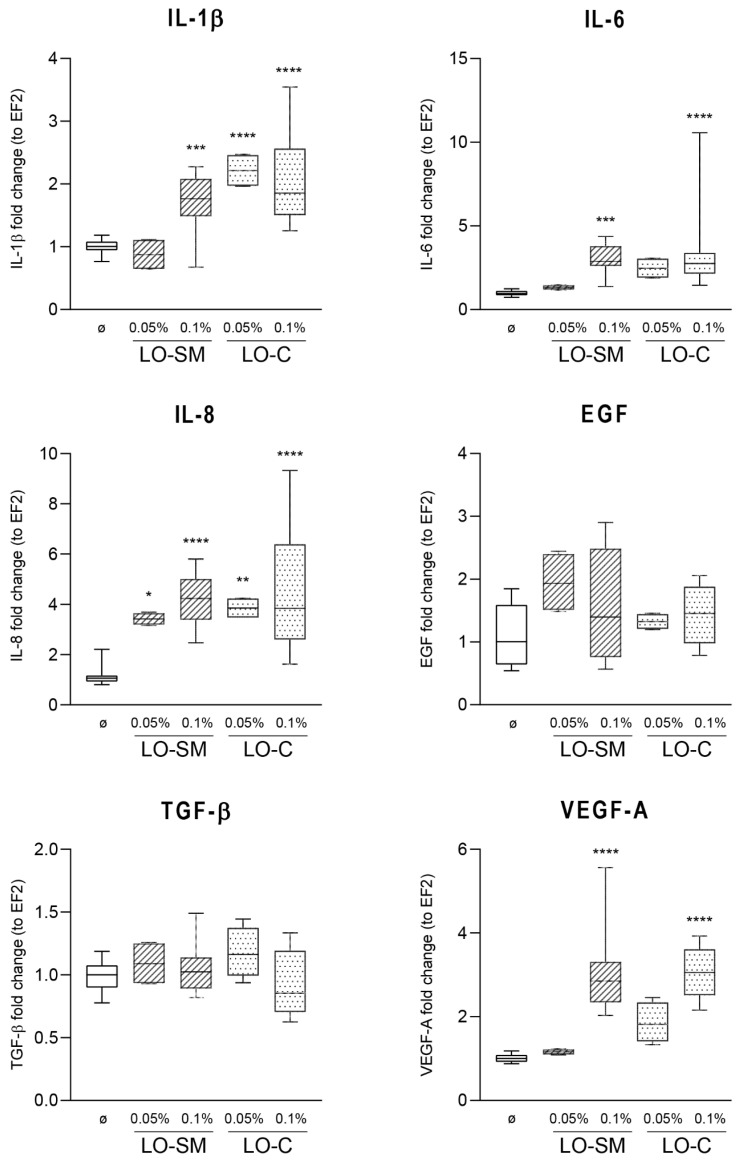
Effect of lavender oil stimulation on the expression level of cytokines in HaCaT cell line. HaCaT cells were stimulated with designated concentrations (*v*/*v*) of commercial or in-house preparation of lavender oil for 4 h. Levels of mRNA production of cytokines: IL-1β, IL-6, IL-8, EGF, TGF-β, and VEGFA were verified with RT-PCR. Values are mean with range. Groups were compared to the control, and statistical analysis was performed using GraphPad Prism and build-in ANOVA test. *p*-values ≤ 0.05 were considered significant. *—*p* = 0.05–0.011; **—*p* ≤ 0.01; ***—*p* ≤ 0.001; ****—*p* ≤ 0.0001.

To determine the influence of lavender oil on the production of proinflammatory cytokines and proregenerative growth factors, gene expression of the selected signaling molecules by HaCaT cells was investigated using real-time PCR. Cells were stimulated with lavender oil (LO-SM and LO-C; 0.1%) for 4 or 24 h, then RNA was isolated, cDNA was transcribed and used as template in RT-PCR. The results revealed significant induction of IL-1β, IL-6, IL-8, and VEGF gene expression upon stimulation with lavender oil. IL-8 expression was enhanced nearly 4-fold. Both LO-SM and LO-C induced a 2-fold increase in IL-6, VEGF, and IL-1β gene expression. At the same time, EGF and TGF-β1 expression was not affected by the presence of the lavender oils (Figure 3).

**Figure 4:**
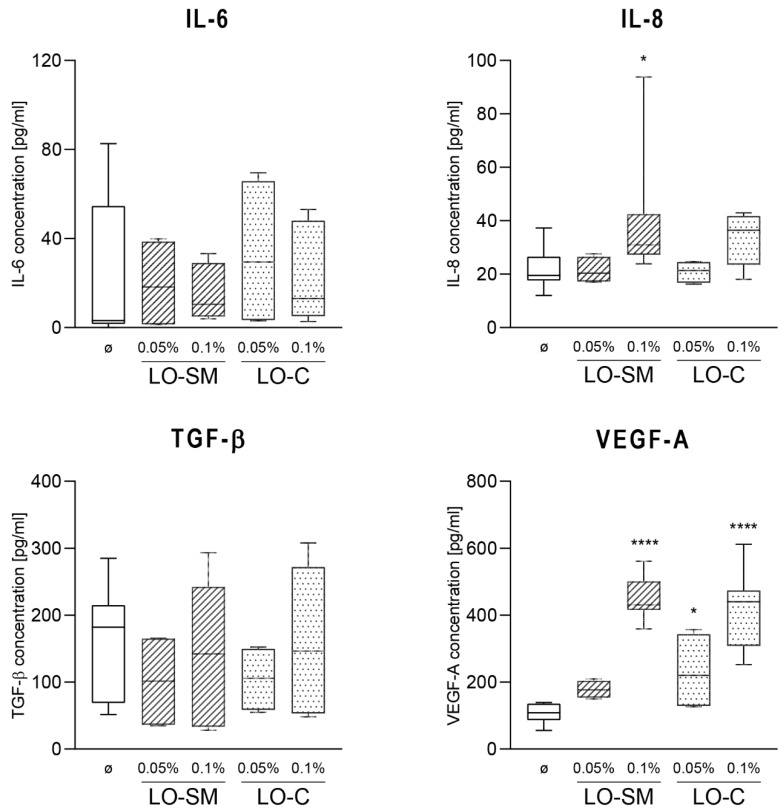
Effect of lavender oil stimulation on the cytokines production in HaCaT cell line. HaCaT cells were stimulated with designated concentrations (*v*/*v*) of commercial or in-house preparation of lavender oil for 24 h. Level of cytokine in cell media: IL-6, IL-8, TGF-β, VEGFA were verified with ELISA. Values are mean with range. Groups were compared to the control, and statistical analysis was performed using GraphPad Prism and build-in ANOVA test. *p*-values ≤ 0.05 were considered significant. *—*p* = 0.05–0.011; ****—*p* ≤ 0.0001.

To evaluate the changes in the HaCaT cytokine secretion to the culture medium, ELISA was performed 24-h post-stimulation. IL-8 levels were increased 50% to the level of 30 pg/mL for LO-SM at 0.1% concentration. A similar trend was observed for LO-C, however, the significance level was not achieved in the statistical analysis when compared to the control cells. This effect was accompanied by the significant increase in VEGF levels observed in medium, which reached 500 pg/mL in cell media treated with 0.1% full oil preparations, observed for both LO-SM and LO-C. The levels of IL-6 or TGF-β were unaffected upon stimulation with any preparation of lavender oil (Figure 4). Similarly, no significant increase in IL-1α and IL-1β concentration in cell culture medium was observed (data not shown). The measured medium concentrations of IL-1α, IL-1β were generally low, indicating the low potential of lavender oil for the proinflammatory stimulation of HaCaT cells.

**Figure 5:**
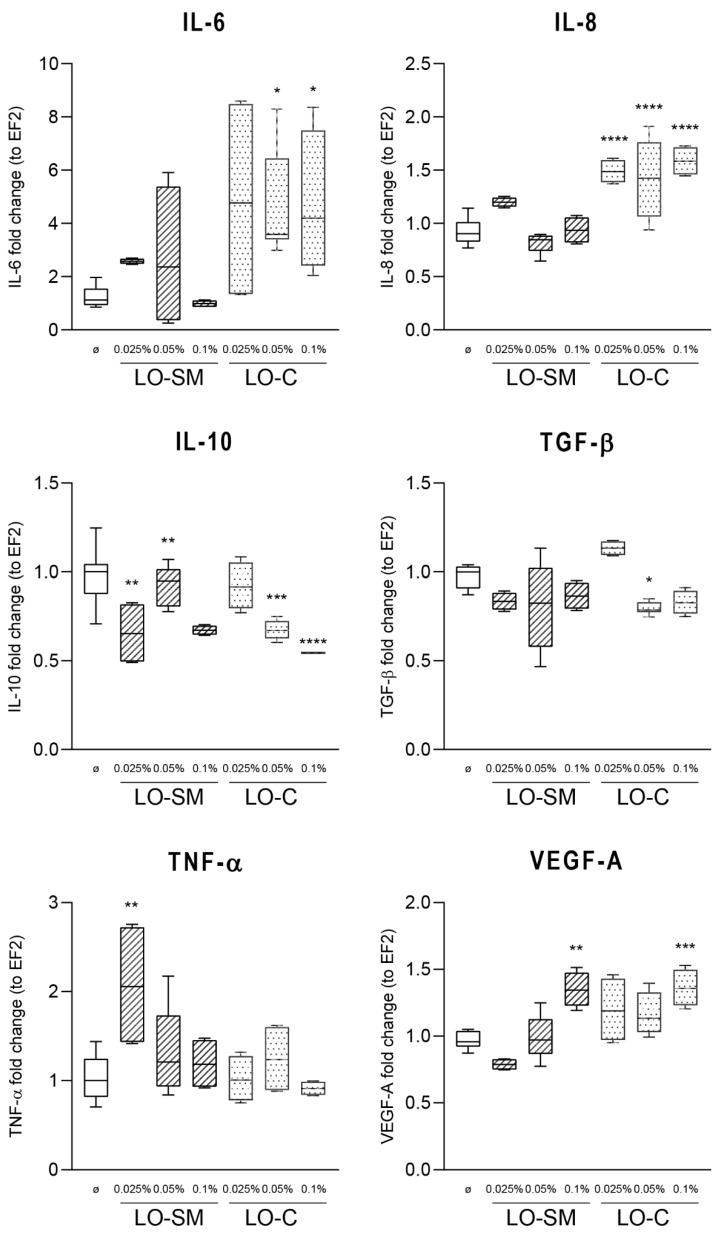
Effect of lavender oil stimulation on the expression level of cytokines in human macrophages. hMDM cells were stimulated with designated concentrations (*v*/*v*) of commercial or in-house preparation of lavender oil for 4 h. Level of mRNA production of cytokines: IL-6, IL-8, IL-10, TGF-β, TNF-α, and VEGFA were verified with RT-PCR. Values are mean with range. Groups were compared to the control, and statistical analysis was performed using GraphPad Prism and build-in ANOVA test. *p*-values ≤ 0.05 were considered significant. *—*p* = 0.05–0.011; **—*p* ≤ 0.01; ***—*p* ≤ 0.001; ****—*p* ≤ 0.0001.

**Figure 6:**
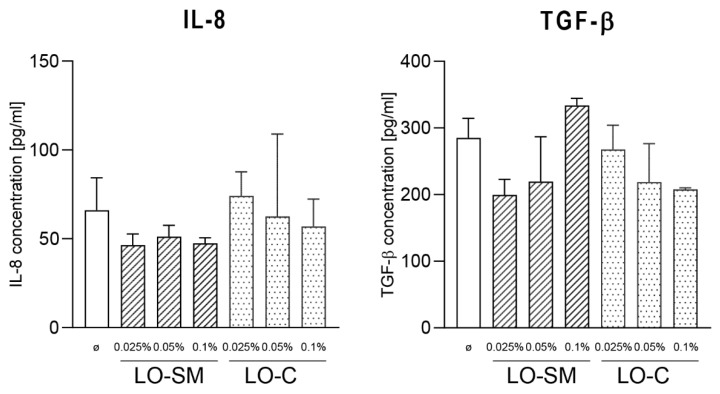
Effect of lavender oil stimulation on the cytokines production in human macrophages. hMDM cells were stimulated with designated concentrations (*v*/*v*) of commercial or in-house preparation of lavender oil for 24 h. Level of IL-6, TGF-β in cell media were verified with ELISA. Values are mean ± SD. Groups were compared to the control, and statistical analysis was performed using GraphPad Prism and build-in ANOVA test. *p*-values ≤ 0.05 were considered significant.

In agreement with this observation, only a limited effect on the cytokine production by human macrophages was observed. hMDMs were stimulated with lavender oil preparations, similarly as described for HaCaT cells, and gene expression of proinflammatory M1-phenotype-related TNF-α, IL-8, IL-6, and proregenerative M2 phenotype-related IL-10 was measured by real-time PCR. Gene expression levels of IL-6 and IL-8 were increased only by LO-C; 7-fold and 2-fold, respectively, when compared to the control. Some induction of TNF-α was observed at the 0.025% LO-SM, yet it was reduced to the control levels at higher concentrations of LO-SM. A weak induction of VEGF-A gene was noticeable, but again, the levels did not reach 2-fold induction. Interestingly, IL-10 expression was reduced ~2-fold, indicating that macrophages remained in M1 phenotype (Figure 5). At the same time, ELISA tests of the cytokine levels in the cell media did not reveal any increase in IL-8 and TGF-β secretion, regardless of timepoint and lavender oil concentration used, indicating the lack of the direct effect of lavender oil preparations on the resting macrophages (Figure 6).

### 2.4. Effect of the Lavender Oil on LPS Stimulated HaCaT Keratinocytes

**Figure 7:**
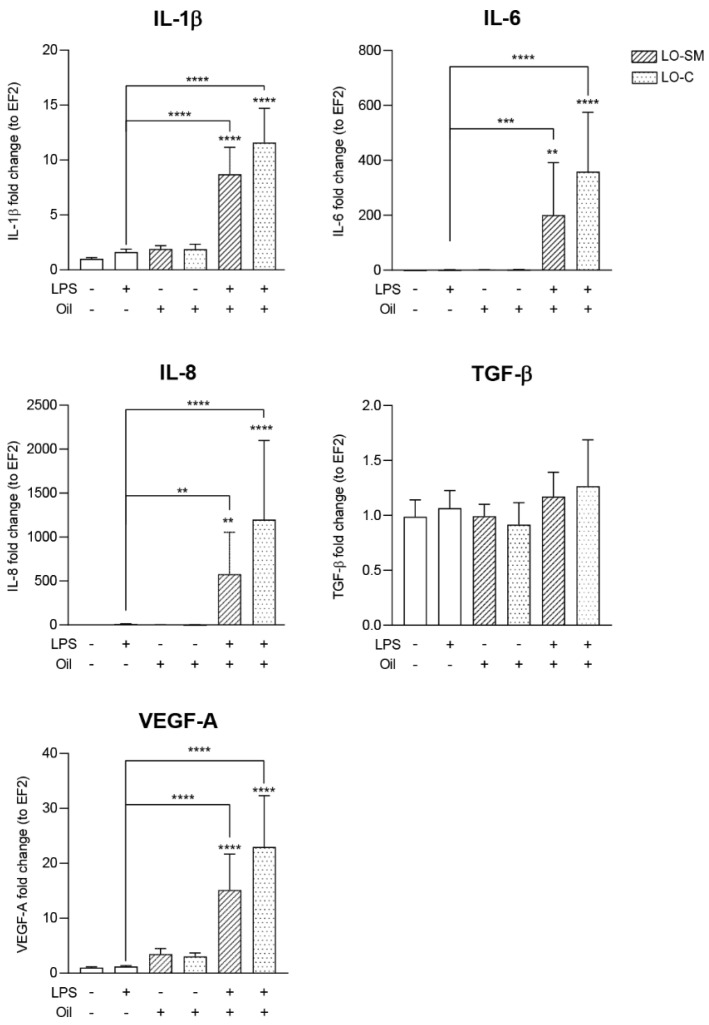
Effect of co-stimulation with lavender oil and bacterial LPS on the cytokines production in HaCaT cell line. HaCaT cells were stimulated with 0.01% of commercial or in-house preparation of lavender oil and 5 ug/mL of *Escherichia coli* LPS for 4 h. Level of mRNA production of cytokines: IL-6, IL-8, TGF-β, and VEGFA were verified with ELISA. Values are mean ± SD. Groups were compared to the control, and statistical analysis was performed using GraphPad Prism and build-in ANOVA test. Individual comparisons between groups are indicated by the gates. *p*-values ≤ 0.05 were considered significant. **—*p* ≤ 0.01; ***—*p* ≤ 0.001; ****—*p* ≤ 0.0001.

To investigate the effect of the lavender oil preparations on the inflammatory-stimulated epithelial and immune cells, we employed the model based on the stimulated HaCaT and macrophage cells. TLR4-interacting ligand, *Escherichia coli* LPS, was used to activate cells prior to the lavender oil application. Co-stimulation of HaCaT cells with LPS and lavender oil resulted in the strong induction of the VEGF-A gene. Both preparations, LO-SM and LO-C, induced VEGF-A expression 3-fold, yet strikingly the presence of LPS exaggerated the effect further, allowing for the 15x (LO-SM) and 23x (LO-C) VEGF-A stimulation. Similarly, IL-1β, IL-6, and IL-8 were synergistically upregulated by co-stimulation with LPS and lavender oil. In contrast, the expression of TGF-β was not affected at all (Figure 7). At the same time, LPS alone did not affect the expression of VEGF nor TGF-β by HaCaT cells.

**Figure 8:**
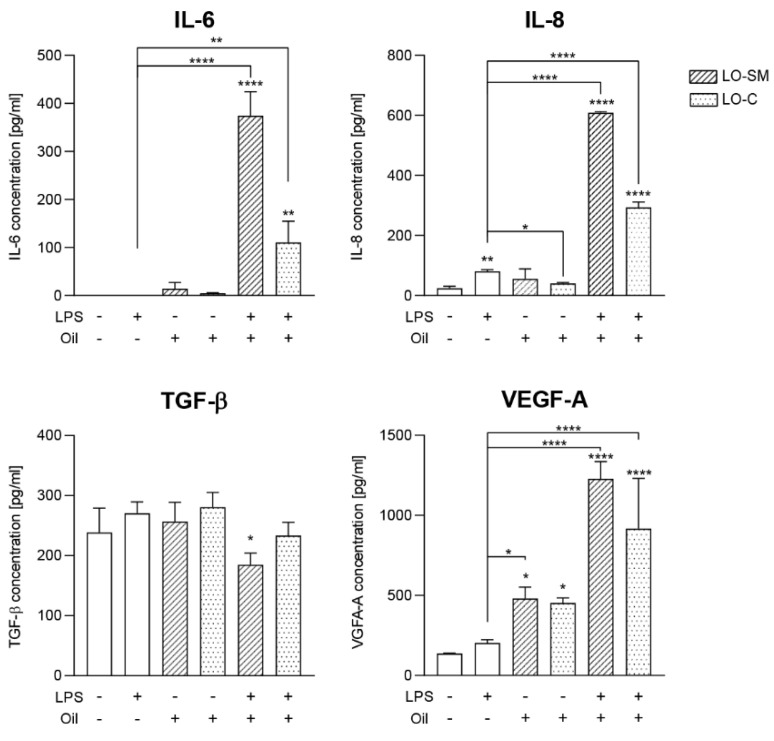
Effect of co-stimulation with lavender oil and bacterial LPS on the cytokines production in HaCaT cell line. HaCaT cells were stimulated with 0.01% of commercial or in-house preparation of lavender oil and 5 ug/mL of *Escherichia coli* LPS for 24 h. Level of cytokine in cell media: IL-6, IL-8, TGF-β, and VEGFA were verified with ELISA. Values are mean ± SD. Groups were compared to the control, and statistical analysis was performed using GraphPad Prism and build-in ANOVA test. Individual comparisons between groups are indicated by the gates. *p*-values ≤ 0.05 were considered significant. *—*p* = 0.05–0.011; **—*p* ≤ 0.01; ****—*p* ≤ 0.0001.

ELISA measurement of the cell medium secreted IL-6 and IL-8 confirmed the synergy between LPS and lavender oil stimulation. Although LO-SM and LO-C stimulated IL-6 and IL-8 production by HaCaT cells, in accordance with the effect observed at the RNA level, in the presence of LPS the effect was strongly enhanced. LO-SM stimulated the release of IL-6 nearly 400-fold, while commercial preparation induced 100× higher concentrations of IL-6 in the medium when compared to the control, reaching nearly 400 pg/mL and 100 pg/mL, respectively. Additionally, IL-8 secretion was synergistically enhanced. When incubated together with LPS, LO-SM and LO-C boosted the IL-8 production by the stimulated cells to 600 (LO-SM) or 300 pg/mL (LO-C), compared to the 40 (LO-SM) and 30 pg/mL (LO-C) induced by the lavender oil alone. Again, LPS alone did not stimulate secretion (IL-6) or induced only a moderate production (50 pg/mL IL-8) of the investigated cytokines by the HaCaT cells. Moreover, the proregenerative response of HaCaT cells was affected as well. Co-stimulation with LPS and lavender oil increased VEGF concentration in the culture medium to 1200 and 1000 pg/mL for LO-SM and LO-C, respectively. This effect, however, was additive rather than synergic, as both LPS and lavender oil alone stimulated VEGF secretion (Figure 8). Collectively, this indicates that lavender oil strongly augments the wound healing response in the activated HaCaT cells, enhancing the effect of the LPS-induced TLR stimulation.

### 2.5. Effects of Lavender Oil on LPS-Prestimulated hMDMs

**Figure 9:**
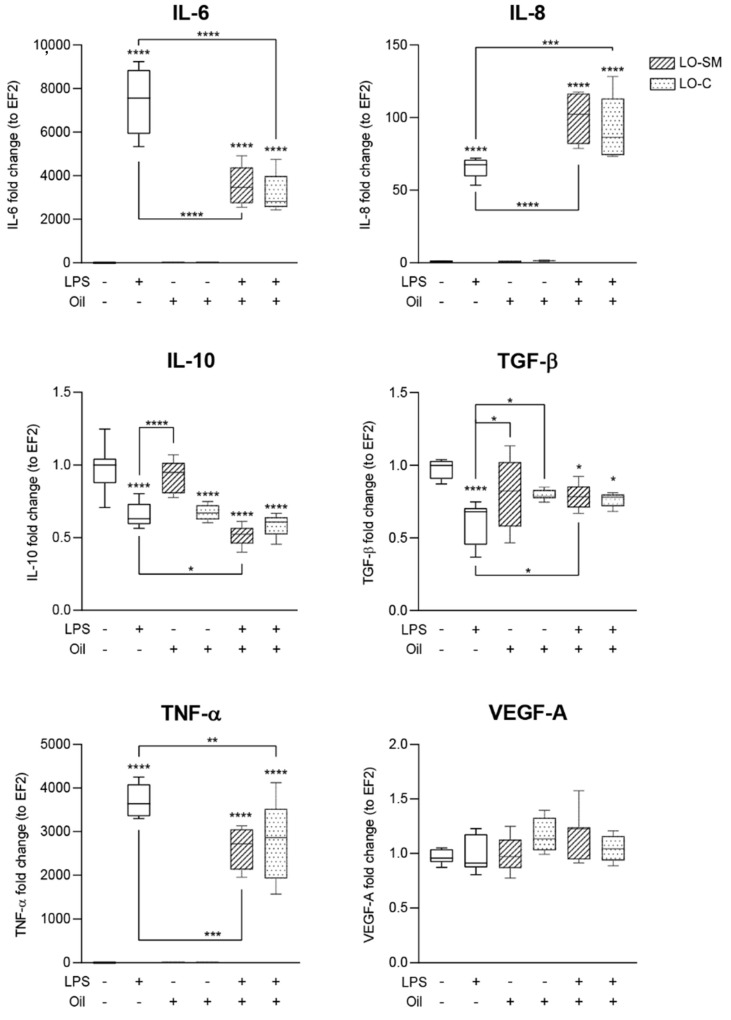
Effect of co-stimulation with lavender oil and bacterial LPS on the expression level of cytokines in human macrophages. hMDM cells were stimulated with 0.05% commercial or in-house preparation of lavender oil and 100 ng/mL of *Escherichia coli* LPS for 4 h. Level of mRNA production of cytokines: IL-6, IL-8, IL-10, TGF-β, TNF-α, and VEGFA were verified with RT-PCR. Values are mean with range. Groups were compared to the control, and statistical analysis was performed using GraphPad Prism and build-in ANOVA test. Individual comparisons between groups are indicated by the gates. *p*-values ≤ 0.05 were considered significant. *—*p* = 0.05–0.011; **—*p* ≤ 0.01; ***—*p* ≤ 0.001; ****—*p* ≤ 0.0001.

Interestingly, the co-stimulation of the cells with the lavender oil and LPS significantly limited the macrophage proinflammatory response. It resulted in a 2500-fold increase in TNF-α (vs. 3500-fold increase during stimulation with LPS alone) and 3000-fold increase in IL-6 expression (vs. 7000-fold increase during stimulation with LPS alone). At the same time, expression of IL-8 by cells stimulated with LPS and lavender oil was additive, reaching 110-fold increase, compared to 80-fold for LPS alone. Both LO-SM and LO-C comparably hindered the proinflammatory gene expression. In parallel, the additive effect on IL-10 production was observed. LPS alone limited IL-10 gene expression by a 30%, while the co-stimulation with either LO-SM or LO-C reduced the IL-10 expression by 50% when compared to the untreated cells. Interestingly, although the LPS-alone lowered the expression of TGF-β, the cells seemed to be protected from the LPS-induced TGF-β production decrease during co-stimulation with lavender oils, retaining levels closer to the control sample, thus displaying a clear anti-inflammatory effect (Figure 9).

**Figure 10:**
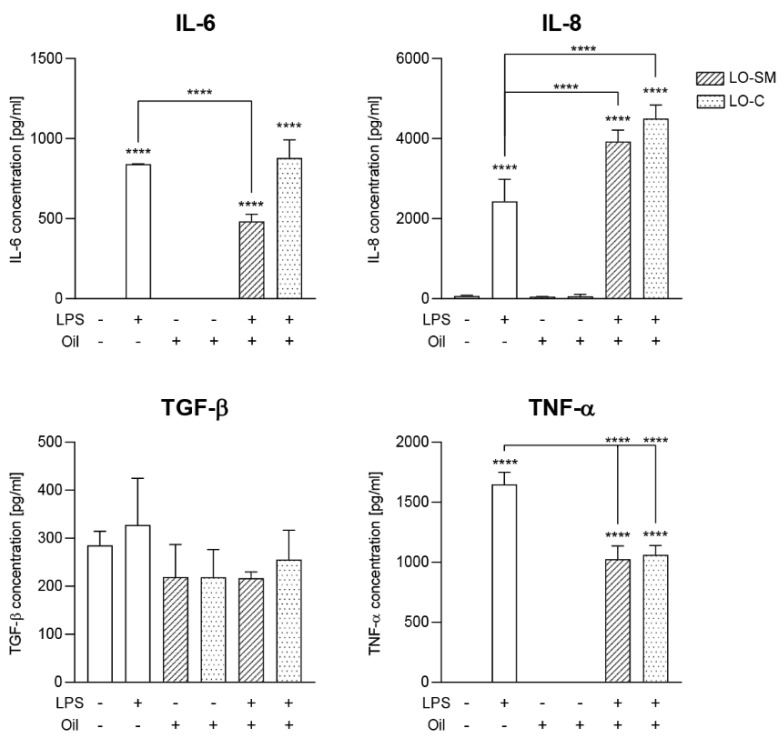
Effect of co-stimulation with lavender oil and bacterial LPS on the cytokines production in human macrophages. hMDM cells were stimulated with 0.05% commercial or in-house preparation of lavender oil and 100 ng/mL of *Escherichia coli* LPS for 24 h. Level of IL-6, IL-8, TNF-α, TGF-β in cell media were verified with ELISA. Values are mean ± SD. Groups were compared to the control, and statistical analysis was performed using GraphPad Prism and build-in ANOVA test. Individual comparisons between groups are indicated by the gates. *p*-values ≤ 0.05 were considered significant. ****—*p* ≤ 0.0001.

Analysis of the cytokine secretion to the cell medium revealed similar trends as observed on the RNA level. LPS induced the IL-6 secretion to 800 pg/mL. However, the limiting effect of lavender oil was observed only with the in-house formulation LO-SM, which resulted in concentrations below 500 pg/mL. TNF-α levels were increased by the LPS stimulation to 1600 pg/mL, while both LO-SM and LO-C lowered the observed TNF-α release to 1000 pg/mL, again indicating the inflammation-limiting effect of lavender oil on human macrophages. At the same time, co-stimulation of IL-8 production by LPS and lavender oil was confirmed, as the LPS co-stimulation with either LO-SM or LO-C resulted in 4000 pg/mL of IL-8 secreted to the medium, compared to 2500 pg/mL for LPS-only. Lavender oil alone nor with LPS pretreatment did not stimulate TGF-β release from macrophages (Figure 10).

## 3. Discussion

### 3.1. Effect of Lavender Oil Origin on the Chemical Composition

The main components of lavender oil are linalyl acetate, linalool, ocimenes, lavandulol, and lavandulyl acetate. Interestingly, the proportions of each appear to be affecting one another, for example, the larger share of linalyl acetate was observed with the smaller content of ocimenes and vice versa [1,14,15,16,17]. The composition of the oils may change due to environmental factors, e.g., the origin of the plant, sunlight exposure, or plant age, as well as plant variety. In many previous works, the chemical composition and antibacterial and antifungal effects of lavender oil have been examined, but different varieties of the same species have not been directly compared.

The chemical composition of the essential oil depends largely on the variety of the narrow-leaved lavender. Adaszyńska et al. [3] analyzed the composition of the oils derived from flowers of different varieties of the narrow-leaved lavender (*Lavandula angustifolia* L.): “Munstead”, “Munstaed Strain”, “Blue River”, “Ellegance Purple”, and “Lavender Lady” cultivated in Szczecin, Poland. The studies showed that different varieties of narrow-leaved lavender differed in individual compounds: from a total of 55 compounds identified, the majority belonged to the group of monoterpenoids and their esters. Sesquiterpenoids were also identified. The authors of the study observed that different varieties of narrow-leaved lavender contained the same main compounds (linalool 15.9–23.9%, linalyl acetate 1.2–4.7%, cis-ocimene 1.1–2.4%, and lavandulol 3.4–4.6%), however, the compounds found in low concentrations were different, which could affect their biological properties.

Kivrak et al. [11] compared the chemical composition and antioxidant effect of six varieties of *L. angustifolia*: “Sevtopolis”, “Yubileina”, “Druzhba”, “Raya”, “Hebar”, “Hemus”, and two varieties of lavandin L. × intermedia (a hybrid of narrow-leaved lavender with broad-leaved lavender): “Grey Hedge” and “Super A” from Turkey. The GC/MS analyses of the essential oils showed 66 components, including linalyl acetate (4.648–46.887%), linalool (28.102–36.801%), β-farnesene (0.914–7.053%), β-caryophyllene (0.637–6.290%), and lavandulyl acetate (0.831–4.840%) as the main components of both species of lavender. Interestingly, there was a significant difference in the content of the main components of the lavender oil (linalyl acetate and linalool) depending on the variety. Hemus variety of *L. angustifolia* contained the highest amount of linalyl acetate content (46.887%), while the highest linalool content (36.801%) was found in lavandin L. × intermedia Super A variety. *L. angustifolia* Sevtopolis variety had the highest content of lavandulyl acetate (4.840%) and β-caryophyllene (6.290%). The concentration of camphor in each *L. angustifolia* variety was below 0.5%, and in L. × intermedia varieties it was over 5%. According to the ISO 3515:2002 standard [5], lavender essential oil contains linalool (25–38%), linalyl acetate (25–45%), and camphor (0.5–1.0%). Lavandin essential oil contains linalool (24–35%), linalyl acetate (28–38%), and camphor (6–8%) according to the ISO 8902:2009 [18].

The oil from *L. angustifolia* has the most variable composition. An example can be a comparison of the composition of the oil depending on the country of origin. In the case of the oil from Bulgaria, the content of ocimenes was in the range of 6.8–7.7%, linalool 30–34%, linalyl acetate 35–38%, and lavandulol and its acetate were not identified. The oil from China did not contain ocimenes and the main components were linalool (24–36%), linalyl acetate (29–36%), lavandulol and its acetate (1.6–1.7%). The oil from India also did not contain ocimenes and the main components were linalool (10%), linalyl acetate (45%), and lavandulol (0.1%) [19,20]. The oil derived from narrow-leaved lavender (*L. angustifolia*) from the varieties which grow in Poland includes in the largest quantities: linalool (15.9−33.8%), linalyl acetate (ca. 21.9%), geraniol (1.4−8.8%), bornyl acetate (ca. 6.1%), and trans-caryophyllene (ca. 6.1%). In smaller amounts, the oil also contained borneol, terpineol, limonene, α-, and β-pinenes. The camphor content was 0.7% [6]. Śmigielski et al. [21] identified seventy-eight compounds in the essential oil obtained from the dried flowers of Lavandula angustifolia, cultivated in Poland. The major constituents of the oil were linalool (30.6%), linalyl acetate (14.2%), geraniol (5.3%), β-caryophyllene (4.7%), and lavandulyl acetate (4.4%).

The subject of our study was the comparison of the composition and proregenerative properties of the narrow-leaved lavender oil that was available on the market and manufactured in Provence, France, with the oil obtained from the narrow-leaved lavender cultivated in Poland, Świętokrzyskie Voivodeship. The composition of the essential oil used for medicinal purposes is subject to the requirements set out in the European and Polish Pharmacopoeia [2,22]. In accordance with these sources, the main components of *L. angustifolia* oil are linalool and its acetate. Both oils studied here met this requirement; however, the self-prepared oil contained less linalool (23.2 vs. 40.2%). This substance is listed in the Regulation (EC) no. 1223/2009 (OJ L 342/59) of the European Parliament and of the Council among the 26 substances causing sensitization. Other substances listed in this regulation and found in the tested oils were geraniol and d-limonene, but their content was very low (<2%). The tested lavender oils had a similar qualitative composition, but they differed in their quantitative composition of individual substances. The obtained results are in line with literature reports [3,6]. However, in the case of self-prepared oil, a much higher content of lavandulyl acetate was found in comparison with the commercial oil (23.2 vs. 5.5%). According to the Pharmacopoeia, its content is usually >0.2%, and for both tested oils, these values were higher.

### 3.2. The Lavender Oil Origin and the Proinflammatory and Proregenerative Cytokine Cellular Response

The wound healing process consists of four overlapping phases—hemostasis, inflammatory, proliferative, and remodeling; their proper regulation is required for fast recovery and minimalization of scar formation. A critical step in the inflammatory phase is the mobilization of immune cells due to the release of DAMPs molecules (danger-associated molecular patterns) from the damaged area and the presence of PAMPs (pathogen-associated molecular patterns) in the case of the typically nonsterile wounds. These factors stimulate the production of signaling molecules (IL-6, IL-8, and VEGF) by epithelial cells [23]. At first, neutrophils infiltrate the area, followed by monocytes, which differentiate into macrophages and begin to perform regulatory functions. Other cell types, including platelets and fibroblasts, take part in extinguishing the inflammatory phase and regulate the course of the proliferative phase.

The in vitro studies focused on the analysis of the responses of epithelial cells (HaCaT human keratinocyte line) and monocyte-derived macrophages. Results showed a concentration-dependent effect of lavender oils on IL-6, IL-8, and VEGF production by keratinocytes markedly increased during co-stimulation of cells with the model PAMP molecule—bacterial LPS. Importantly, the levels of those cytokines were significantly higher in cells co-treated with in-house produced oil. Interleukin-6 is the central cytokine of the wound healing reaction. It is responsible for the regulation of the inflammatory phase and further transition to a proliferative state. It is produced in the early stages in response to tissue damage and induces auto- and paracrine inflammatory reaction in keratinocytes, macrophages and fibroblasts. Later in the proliferative stage, the presence of IL-6 is responsible for the change in macrophage polarization from proinflammatory (M1) to proregenerative (M2) phenotype, migration and proliferation of keratinocytes, the increase in VEGF production and induction of TGF-β production by fibroblasts. Finally, decreasing amounts of IL-6 are indicative of the late remodeling stage of wound healing [24].

The induction of IL-6 expression upon lavender oil stimulation shown in this manuscript may be interpreted as fundamental for promoting wound healing in epithelial tissue, and it is consistent with the previously observed reaction of keratinocyte cell lines [25,26]. Indeed, in vivo mice models identified IL-6 as indispensable in tissue regeneration. IL-6 knock-out BALB/c mice show prolonged wound healing due to decreased TGF-β and VEGF production in tissues [27], while the supplementation with recombinant IL-6 restored homeostatic wound healing responses [28]. Moreover, reports indicate insufficient production of IL-6 in diabetic and persistent human wounds [27].

As mentioned above, IL-6 induces the production of TGF-β in fibroblasts—a step crucial to their regenerative proliferation, differentiation to myofibroblasts and formation of the granular tissue. In accordance, in vivo rat model examining the effect of the lavender oil on the healing of burn wounds indicated an enhanced TGF-β production, accompanied by the higher rate of fibroblast proliferation, increased production of collagen and faster wound healing [29].

IL-8, an effector molecule of IL-6 [30,31,32], serves as the main chemoattractant to neutrophils in wound exudative fluid, as demonstrated in mice models. Moreover, it possesses stimulating potential for keratinocyte proliferation in vitro and improves wound healing potential in an in vivo model. IL-8 was also found to promote vascularization, supplementing reduced levels of typical proangiogenic factors in non-healing wounds [33]. Although some reports indicate prolonged chemoattractant activity of IL-8 as promotor of the excessive ECM degradation via increased neutrophil influx [34], others shown, that the levels of IL-8 increased with the progress of wound healing and exerted a positive, proangiogenic effect, not related to activation of neutrophils at later stages of healing [35]. Interestingly, heightened production of the IL-8 was also observed in our study, most notably during co-stimulation of cells with LPS.

Finally, we have identified the potential of the lavender oil to increase the production of VEGF. Several studies indicate the importance of VEGF in wound healing, as the neutralizing antibodies, low molecular weight signal transduction blockers, or induced genetic deletions, reducing VEGF activity resulted in delayed wound healing [36]. In particular, the in vivo model of VEGF-deficient keratinocytes hampered the formation of new blood vessels and slower wound healing [37]. Interestingly, lowered VEGF levels and poor angiogenesis are observed in difficult to heal diabetic wounds [38], and VEGF supplementation accelerates wound closure, granular tissue formation and improves the mechanical properties of the healed area [39,40]. VEGF has been shown to influence the proliferation and migration of keratinocytes, both in vitro [41,42] and in vivo [43,44,45]. In addition, VEGF promotes keratinocyte survival in the UV irradiation model [45,46]. At the same time, VEGF promotes macrophage polarization and the proregenerative phenotype of M2 [47], while the reduced levels of VEGF correlate with the predominance of the proinflammatory phenotype of M1 and reduced phagocytosis of apoptotic neutrophils in diabetic wounds [48,49].

The presence of lavender oil preparations did not affect macrophage functions without bacterial LPS co-stimulation. In the presence of the endotoxin, however, the expected proinflammatory response was hampered by lavender oil. As expected, both the expression of the TNF-α, IL-6, and IL-8 genes, and the levels of cytokines released into the medium were increased by LPS alone. Our in-house preparation of lavender oil alleviated LPS-induced inflammatory response, causing about 50% reduction in the expression of TNF-α and IL-6 genes and was superior in the reduction of IL-6 when compared to the commercial lavender oil. Since hMDMs, as opposed to HaCaT cells, are considered to be specialized immune response cells, any reduction in their proimmune response would strongly limit the excessive activation of all the immune cells and, therefore, facilitate the return to homeostasis. This, in turn, will be beneficial during the proliferative phase of wound healing, resulting in a quicker and complication-free wound closure.

## 4. Materials and Methods

### 4.1. Plant Material

Whole plant material of *Lavandula angustifolia Mill*. cultivars including “Hidcote Blue”, “Nana Alba”, and “Dwarf Blue” were collected from Słupia Nadbrzeżna in Poland by Ostrowieckie Centrum Medyczne civil partnership, in June 2019 when the crop was full of blossom. The plant materials were studied fresh.

### 4.2. Isolation of Lavandula Angustifolia Essential Oil

The distillation study was conducted using 250 g of dried lavender flowers. Freshly harvested parts of plants were immediately subjected to steam distillation for 2 h to extract the essential oil. The lavender oil (LO-SM) was separated from the hydrolate, dried with anhydrous sodium sulphate, and stored in an amber bottle at +4 °C in a refrigerator until time of analysis.

The commercially available oil (LO-C) obtained from lavender grown in Provence, France, was used as the control sample.

### 4.3. GC/MS Analysis

#### 4.3.1. Chemicals and Reagents

Dichloromethane was obtained from Avantor Performance Materials Poland S.A. company (purity 99.8%). The standard substances (linalool, linalyl acetate, lavandulyl acetate, (-)-bornyl acetate, (-)-trans-caryophyllene, and geraniol) had a purity of 93.0 to 99.3% and were purchased from Sigma-Aldrich Sp. Z o.o. (Poznan, Poland). Two lavender oils were analyzed: the first one was our in-house preparation, developed at the Cracow University of Technology (LO-SM), and the second was commercially available lavender oil (LO-C).

#### 4.3.2. Preparation of Standards and Samples

Identification analyses were carried out for 1% samples of the examined oils dissolved in dichloromethane. For quantitative analyses, 50 mg of the analyzed oils dissolved in 1 mL of dichloromethane were used, which was then diluted 20 times before injecting into the chromatograph. Calibration curves for each substance were performed using standard analysis in the concentration range of about 50–4000 µg/mL in dichloromethane. The ranges were modified depending on the concentrations of substances in the tested samples.

#### 4.3.3. Apparatus and Chromatographic Conditions

An Agilent Technology 6890N gas chromatograph with a 5973 MSD mass spectrometer was used. Capillary column INNOWax (60 m × 0.25 mm i.d., film thickness 0.5 μm) was installed in the gas chromatograph coupled into the ion source of the mass spectrometer (Agilent 5973 MSD). The column worked in programmed conditions from 50 °C (for 2 min), then temperature increased 5 °C/min to 80 °C (0 min), 10 °C/min to 120 °C (0 min), 20 °C/min to 240 °C, and the analysis temperature was maintained up to 30 min. The gas (helium) flow through the column was constant at 25 cm/s. The injector temperature was 250 °C, and the volume of injected sample was 1 µL in split mode (20:1) for identification and 50:1 for quantitative analysis. The mass spectrometer (Agilent 5973 MSD) worked in scan mode with electron ionization (EI) with 70 eV energy. The range of measured masses was 10–350 *m/z*.

#### 4.3.4. Method Validation

The specificity of the analytical method has been ensured by the use of a mass spectrometer and the comparison of the obtained mass spectra of individual substances with the NIST MS Search 2.0 standard library, and in the quantitative analysis, additionally using the high purity analytical standards of substances tested. The quantification method was validated for linearity and correlation coefficient, precision, limit of quantification (LOQ) and limit of detection (LOD).

### 4.4. Biological Activity of Lavender Oils

#### 4.4.1. Cell Culture

HaCaT cells were cultured in Dulbecco’s Modified Eagle’s Medium (DMEM) (ThermoFisher, Life Technologies, Warsaw, Poland)) containing 10% FBS (ThermoFisher), 100 U/mL penicillin (ThermoFisher), and 100 ug/mL streptomycin (ThermoFisher) in 37 °C/5% CO_2_. Prior to each experiment, the medium was changed to DMEM without any supplementation.

#### 4.4.2. MTT

HaCaT cells were seeded on 96-well plates (50,000 cells/well) a day prior to the experiment. Cells were incubated with various lavender oil formulations at concentrations ranging 0–25%. After 4 and 24 h, the medium was removed, cells were incubated with 200 uL/well of 0.5 mg/mL MTT (Thiazolyl Blue Tetrazolium Bromide) (Sigma-Aldrich) in DMEM for up to 20 min 37 °C/5% CO_2_. The medium was removed again, and formazan crystals were dissolved in 120 uL of isopropanol acidified with 5 mM hydrochloric acid (POCH, Gliwice, Poland). 90 uL of samples from each well were transferred to a new, transparent 96-well plate. The absorbance of samples was measured using Spectra Max Gemini EM (Molecular Devices) at 570 nm. Results were calculated as a percentage of lavender oil-treated cells compared to the untreated control (cells in DMEM medium).

#### 4.4.3. Isolation of Monocyte-Derived Macrophages from Human Peripheral Blood

Whole blood donated by anonymous donors at the Regional Blood Donation and Haemotherapy Centre (Kraków, Poland) was centrifuged (400 g, 12 min, 20 °C). Plasma was collected, heat-inactivated (water bath, 30 min, 56 °C), centrifuged (3000 g, 15 min, 20 °C) and stored as autologous plasma for medium supplementation during cell culture. Meanwhile, blood morphotic elements were resuspended in PBS and applied on Percoll (density 1077 g/mL, Sigma Aldrich, St. Louis, MO, USA). After centrifugation (400 g, 35 min, RT) buffy coats (formed between the plasma and Percoll layers) were collected and suspended in RPMI 1640 medium (ThermoFisher) with 1% FBS. Cells were centrifuged (350 g, 12 min, 4 °C) and supernatant was discarded. Cells were resuspended in 6 mL of RPMI with 1% FBS, gently applied on 1.5 mL of FBS and centrifuged (280 g, 10 min, 4 °C). Finally, cells were resuspended in RPMI 1640 medium supplemented with 10% autologous HI plasma and 50 μg/mL gentamicin (Thermo Scientific) and seeded at density of 3 mln cells/well on 24-well plates. After 24 h of culture, non-adherent cells were removed by PBS washes. Remaining adherent cells (approx. 300,000 cells/well) were cultivated in fresh medium (RPMI 1640 medium with 10% autologous HI plasma, gentamicin) and allowed to differentiate to monocyte-derived macrophages (hMDMs) for at least 7 days, with medium changed every 2 days.

Human blood from healthy donors for HMDM isolation was obtained from Red Cross, Krakow, Poland, as described in [50]. The Red Cross de-identified blood materials as appropriate for the confidentiality assurance of human subjects. Donors provided written informed consent for the collection of samples and subsequent cell isolation and analysis. Thus, this study adheres to appropriate exclusions from the approval of human subjects, according to current regulations.

#### 4.4.4. Effect of Lavender Oils on Eukaryotic Cells (HaCaT and hMDM)

Confluent cultures of HaCaT cells and fully differentiated hMDM cells (200 or 300 thousand cells respectively per well of a 24 well plate) were stimulated with different concentrations of lavender oil. In an alternative setup, cells were prepared in the same manner, but co-stimulated with *Escherichia coli* serotype O111:B4 LPS (SigmaAldrich) (concentration for HaCaT: 5 µg/mL, concentration for hMDM: 100 ng/mL) diluted in culture medium without supplements before the addition of the lavender oil preparations. After 4 and 24 h, cell media were collected, cells were washed with PBS and lysed with TRIzol reagent (ThermoFisher).

#### 4.4.5. RNA Isolation, Reverse Transcription, and Quantitative Real-Time PCR

RNA was isolated from TRIzol reagent lysates as specified by the manufacturer. Next, equal amounts of RNA were reverse transcribed using High Capacity Reverse Transcription Kit (ThermoFisher). cDNA was mixed with primer pairs (Table 3) in the final concentration of 0.33 µM and the PowerUp SYBR Green MasterMix (ThermoFisher) reagent. Reactions were run in the following conditions: 5 min 95 °C; 40 cycles of: 30 s 95 °C, 30 s 58 °C, and 30 s 72 °C; final elongation step was followed with melting curve analysis for product evaluation. The fold change of gene expression was calculated using the ddCt method, compared to the EF2 expression.

#### 4.4.6. ELISA

Cytokine levels (IL-1α, IL-1β, IL-6, IL-8, TGF-β1, VEGF, and TNFα) in the cell culture media were measured using commercial ELISA kits (R&D) according to the manufacturer’s instructions. In short, MaxiSorp 96-well plates (Thermo Scientific) were coated overnight with appropriate coating antibodies diluted in PBS. Next, the wells were washed with washing buffer (0.05% Tween in PBS) and blocked for 1 h RT with 200 µL of 1% BSA diluted in PBS. The wells were washed and 100 µL of samples were added to the wells and incubated for 2 h in RT. After a thorough wash, wells were incubated with the primary antibodies conjugated with biotin for 2 h and washed again. Then, the wells were incubated with HRP-conjugated streptavidin for 20 min. Prior to developing, the wells were washed five times, and 100 µL of TMB substrate solution (TMB Substrate Reagent Set, BD) was added per well. After the signal was developed sufficiently, the reaction was stopped with 50 µL 2N H_2_SO_4_ (POCH). The absorbance was measured at 450 nm and 570 nm.

## 5. Conclusions

Previous reports showed the wound healing potential of *Lavandula angustifolia* essential oil. Herein we postulate that the lavender oil mechanism of action and effectiveness depend on its composition and, therefore, origin and growth conditions of the plant.

The presented in vitro studies focused on the analysis of the responses of epithelial cells (HaCaT human keratinocyte line) and human monocyte-derived macrophages. Results showed the concentration-dependent induction of production of IL-6 and IL-8 by keratinocytes markedly increased during co-stimulation of cells with the bacterial LPS. Inflammatory reaction was fine-tuned, as TNF-α production in hMDM was limited in the presence of lavender oil in the LPS-stimulated macrophages. Additionally, the proregenerative response of HaCaT cells potentiated by the VEGF cytokine was induced as well.

Reported results indicate lavender oil has a strong potential to enhance the local, tissue-derived proinflammatory and proregenerative response, while simultaneously limiting the inflammatory stimulation of the immune system cells, with in-house preparation performing significantly better in the in vitro cell models. This effect may be attributed to the increased concentration of lavandulyl acetate and decreased linalool acetate in the in-house preparation. Further studies are required to delineate the role of the VEGF stimulation and inflammatory modulation in the lavender oil-supported wound healing and to validate the importance of the individual lavender oil components.

## Figures and Tables

**Table 1:** Validation parameters for the determination of test substances.

No	Substance	Range (µg/mL)	Linear Regression	r	Precision (%)	LOD (µg/mL)	LOQ (µg/mL)
1	Linalool	487 ÷ 3893	y = 1321.3x + 876850	0.9997	1.3	1.18	3.92
2	Linalyl acetate	513 ÷ 4108	y = 1789.9x + 830995	0.9996	0.9	0.99	3.30
3	Lavandulyl acetate	107 ÷ 1074	y = 838.52x + 42043	0.9997	2.6	1.54	5.12
4	(–)-Bornylu acetate	49 ÷ 487	y = 3751.4x + 68284	0.9995	1.8	0.69	2.30
5	(–)-*trans*-Caryophyllene	50 ÷ 501	y = 2597.3x + 8671.5	0.9996	1.2	1.01	3.36
6	(–)-Geraniol	49 ÷ 491	y = 3062x + 8924.9	0.9996	0.6	0.79	2.62

r—Correlation coefficient; LOD—limit of detection; LOQ—limit of quantification.

**Table 2:** Percentage (*w/w*) of individual substances in the analyzed oil samples.

No	Substance	Percentage (%)	
		LO-C	LO-SM
1	Linalool	40.2	23.2
2	Linalyl acetate	44.0	40.6
3	Lavandulyl acetate	5.5	23.2
4	(–)-*trans*-Caryophyllene	1.8	2.3
5	(–)-Geraniol	0.6	1.1

**Table 3:** Oligonucleotides used for real-time PCR.

Gene	Forward (5′ » 3′)	Reverse (5′ » 3′)
EF2	GACATCACCAAGGGTGTGCAG	TTCAGCACACTGGCATAGAGGC
EGF	AATAGTGACTCTGAATGTCC	GCGCAGTTCCCACCA
TGFβ1	CACCCGCGTGCTAATGG	ATGCTGTGTGTACTCTGCTTGAACT
VEGF-A	CGGTGTCTGTCTGTGTGTC	AAGAGGAAAGAGGTAGCAAGAG
IL1β	CCACAGACCTTCCAGGAGAATG	GTGCAGTTCAGTGATCGTACAGG
IL6	CAGGAGCCCAGCTATGAACT	GAAGGCAGCAGGCAACAC
IL8	AGACAGCAGAGCACACAAGC	AGGAAGGCTGCCAAGAGAG
IL10	TCCTTGCTGGAGGACTTTAAGGGT	TGTCTGGGTCTTGGTTCTCAGCTT
TNFα	GTCAGATCATCTTCTCGAACCCCGA	CAGGGCAATGATCCCAAAGTAGA

## Data Availability

The data presented in this study are available in this article.

## Sample Availability

Samples of the compounds are available from the authors.
